# The Evolving Role of Coronary Artery Imaging in Assessing Cardiovascular Risk from Lipoprotein(a)

**DOI:** 10.1007/s11883-026-01440-0

**Published:** 2026-07-10

**Authors:** Michael T. Savides, Harpreet S. Bhatia, Inna Kuznetsova, Michael J. Wilkinson

**Affiliations:** 1 Tufts University School of Medicine, Boston, Massachusetts USA; 2 Division of Cardiovascular Medicine, Department of Medicine, University of California San Diego, 9434 Medical Center Dr., Mail Code 7241, La Jolla, CA 92037-1300 USA; 3 Sargent College of Health and Rehabillitation Sciences, Boston University, Boston, Massachusetts USA

**Keywords:** Lipoprotein(a), Lp(a), Vascular imaging, Coronary angiography, Calcium scoring, Risk stratification

## Abstract

**Purpose of Review:**

Summarize current literature on the use of coronary artery imaging modalities in examining the atherosclerotic and arterial inflammatory manifestations of elevated lipoprotein(a) [Lp(a)]. Evaluate the evolving role of coronary imaging in risk stratification and treatment decisions.

**Recent Findings:**

While Lp(a) and calcium scoring (CAC) are independently associated with cardiovascular risk, Lp(a) is associated with CAC incidence and progression. Lp(a) is associated with greater plaque prevalence, multivessel disease, and high-risk morphologies, while also being linked to accelerated progression of mixed and lipid-rich plaque by coronary CT angiography. Intravascular imaging modalities further show associations between Lp(a) and thin-cap fibroatheroma, lipid-rich plaque, percent atheroma volume, and plaque progression.

**Summary:**

Lp(a) is associated with non-calcified or mixed coronary plaque morphology and progression, as well as increased risk of high-risk plaque features. While additional research is needed, current literature suggests that coronary imaging may be an important tool in cardiovascular risk stratification for individuals with elevated Lp(a).

**Graphical Abstract:**

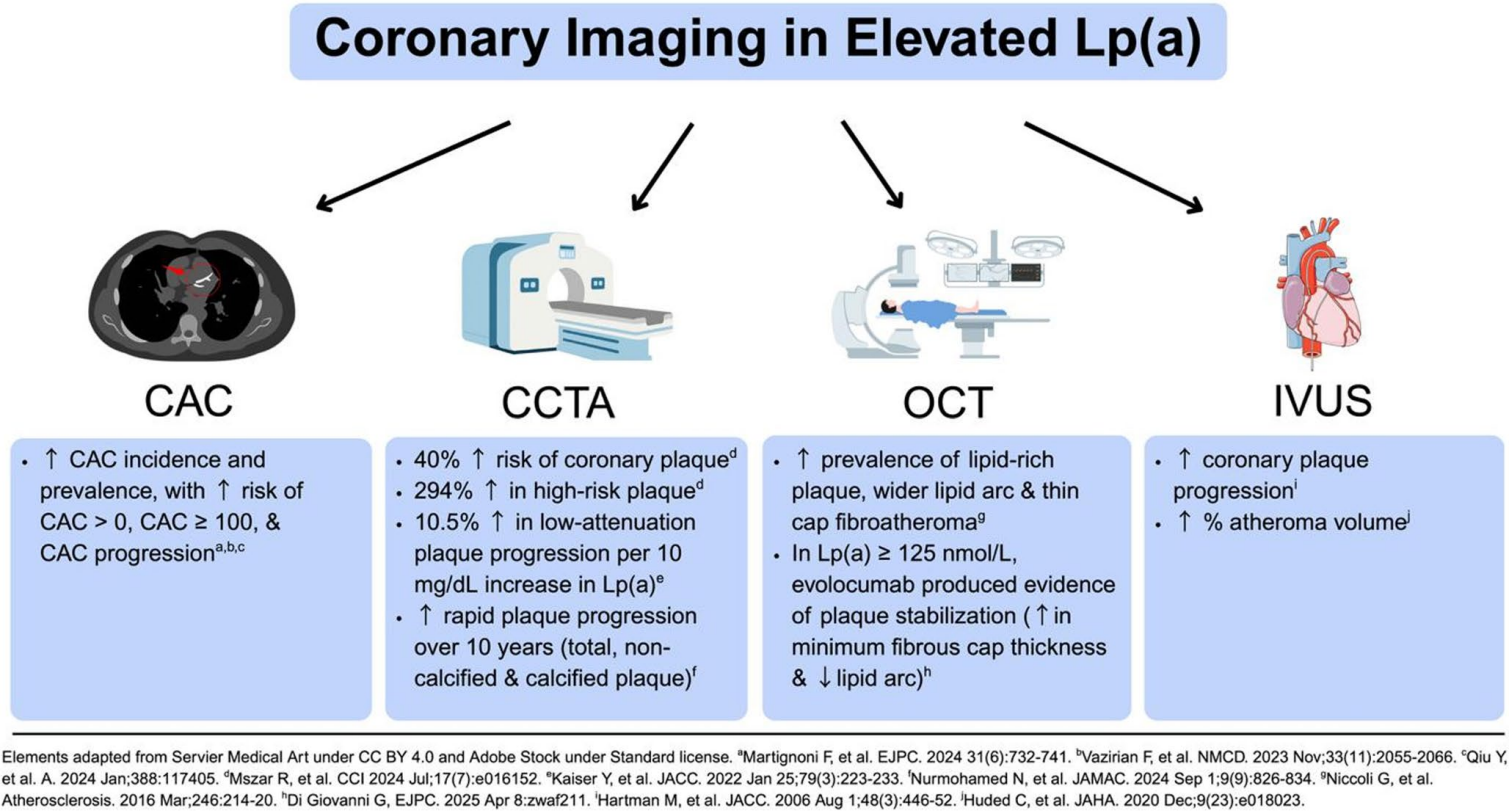

## Introduction

Lipoprotein(a) [Lp(a)] is an inherited, causal risk factor for multiple forms of cardiovascular disease, including atherosclerotic cardiovascular disease (ASCVD) and calcific aortic valve stenosis [1, 2]. Lp(a) is structurally similar to low-density lipoprotein (LDL), with an added apolipoprotein(a) covalently linked to apolipoprotein B-100 by a disulfide bond. However, Lp(a) levels are tied to genetics to a greater extent than LDL in most individuals, with heritability estimates of approximately 70–90%. Additionally, there appears to be minimal influence from environmental or lifestyle factors, and single nucleotide polymorphisms in *LPA* account for most individual variation in plasma Lp(a) levels [3]. Therefore, multiple national organizations recommend obtaining an Lp(a) level in every adult at least once in their lifetime [4–7]. Most current consensus statements from professional societies use a threshold of Lp(a) ≥ 50 mg/dL (≥ 125 nmol/L) as high risk [1, 7, 8].

Evidence from epidemiological studies, genome-wide association studies, and Mendelian randomization supports a causal link between elevated Lp(a) and the development of ASCVD [9–11]. The pathophysiology behind this link has been shown to be multifactorial. Lp(a) promotes atherogenesis by entering the arterial intima, delivering oxidized phospholipids (OxPLs), and ultimately accelerating foam cell formation, plaque development, and vascular wall remodeling [12]. Additionally, the apo(a) component structurally resembles plasminogen, allowing Lp(a) to competitively inhibit fibrinolysis and promote thrombosis [13].

The relationship between Lp(a) and imaging characteristics of atherosclerosis and arterial inflammation has been widely investigated using computed tomography, ultrasound, magnetic resonance, nuclear medicine, and invasive angiography (Table 1). Recent reviews and editorials have provided high-quality analyses of Lp(a) that consider the unique vascular imaging characteristics of individuals with elevated Lp(a). Here we seek to expand and summarize the growing understanding of how individuals with elevated Lp(a) may be at increased risk for higher risk forms of atherosclerosis, detected through both non-invasive and invasive techniques [14–19]. 

**Table 1:** Select studies evaluating the relationship between Lp(a) and findings from coronary artery imaging modalities

Author	Year	Key Findings
CAC Scoring		
Vazirian, et al. [36]	2023	In asymptomatic patients with known cardiovascular disease, the pooled odds ratio for CAC incidence comparing the top versus the bottom tertile of baseline Lp(a) levels was 1.58 (95% CI, 1.38–1.80)
Martignoni, at al. [37]	2024	In asymptomatic patients without known cardiovascular disease, elevated Lp(a) was associated with increased odds of CAC > 0 [OR 1.31; 95% CI 1.05–1.64; *P* = 0.02], CAC ≥ 100 (OR 1.29; 95% CI 1.01–1.65; *P* = 0.04), and CAC progression (OR 1.43; 95% CI 1.20–1.70; *P* < 0.01)
Qiu, et al. [38]	2024	Among all patients, elevated Lp(a) was associated with higher prevalence of CAC both as a categorial variable (OR, 1.31; 95% CI, 1.06 to 1.61; *P* = 0.01) and a continuous variable (OR, 1.05; 95% CI, 1.02 to 1.08; *P* = 0.003). Elevated Lp(a) also associated with greater CAC progression (OR, 1.54; 95% CI, 1.23 to 1.92; *P* = 0.0002)
Bhatia, et al. [41]	2026	Among those with CAC = 0, incidence of ASCVD was higher in those with Lp(a) > 50 mg/dL compared to those with Lp(a) ≤ 50 mg/dL (4.9 vs. 3.8/1,000 person-years, HR: 1.28; 95% CI: 1.01–1.60), although absolute event rates remained low (HR per SD: 1.13; 95% CI: 1.06–1.21; HR for Lp(a) > 50 mg/dL: 1.32; 95% CI: 1.12–1.55). An association between Lp(a) and increased risk was also present in patients with CAC > 0 (21.2 vs. 18.2/1,000 person-years, HR: 3.03; 95% CI: 2.52–3.64), with the greatest overall risk found in those with both CAC ≥ 300 and Lp(a) > 50 mg/dL (HR: 6.12; 95% CI: 4.80–7.81)
CCTA		
Kaiser, et al. [54]	2022	Lp(a) was associated with progression of LAP over 12 months (β = 10.5% increase for each 50 mg/dL Lp(a), 95% CI: 0.7%-20.3%)
Mszar, et al. [45]	2024	In fully adjusted models, elevated Lp(a) was associated with the presence of any plaque (OR, 1.40 [95% CI, 1.05–1.86]) and ≥ 2 high-risk plaque features (OR, 3.94 [95% CI, 1.82–8.52]). Among those with a CAC score of 0, a higher percentage of any plaque was found in participants with elevated Lp(a) (24.2% versus 14.2%; *P* < 0.001)
Nurmohamed, et al. [55]	2024	Lp(a) was associated with more rapid progression of plaque burden over 10 years, as well as overall plaque volume, for total, non-calcified, and calcified plaque
Fathieh, et al. [46]	2025	Elevated Lp(a) linked to mixed calcified and non-calcified plaque morphology (β = 4.75, *P* = 0.001), but not either type individually
OCT		
Niccoli, et al. [59]	2016	Elevated Lp(a) associated with higher prevalence of lipid-rich plaque (67% vs. 27%; *P* = 0.02), wider lipid arc (135 ± 114 vs. 59 ± 111; *P* = 0.03), and prevalence of TCFA (38% vs. 10%; *P* = 0.04)
Muramatsu, et al. [60]	2019	Increased prevalence of TCFA in those with high Lp(a) compared to those with low Lp(a) (23% [*n* = 20] vs. 11% [*n* = 19], *p* = 0.014). Similar prevalence of TCFA between Lp(a) groups among participants with low LDL-C, but significantly higher prevalence of TCFA in higher Lp(a) group among those with high LDL-C (39% [14/36] vs. 10% [5/50], *P* = 0.001)
Di Giovanni, et al. [62]	2025	Among patients with Lp(a) ≥ 125 nmol/L, evolocumab therapy led to lower LDL-C levels (21.7 ± 10.3 vs. 94.5 ± 22.9 mg/dL; *P* < 0.001), lower Lp(a) levels (156.0 (136.0, 187.0) vs. 204.0 (170.5, 290.5) nmol/L; *P* = 0.007), changes in minimum FCT (+ 51.6 ± 40.9 vs. +12.4 ± 23.9 μm; *P* < 0.001), and changes in lipid arc (− 60.9 ± 56.5° vs. −9.1 ± 70.8°; *P* = 0.008) compared to placebo
IVUS		
Hartmann, et al. [66]	2006	Positive correlation was demonstrated between Lp(a) and changes in plaque-plus-media area (*r* = 0.58; *P* < 0.0001)
Huded, et al. [67]	2020	Elevated Lp(a) group had significantly higher percent atheroma volume in risk-adjusted analyses (38.7% ± 0.5 versus 37.5% ± 0.5, *P* < 0.001). Also found a significant association of increasing risk-adjusted percent atheroma volume across Lp(a) quintiles (*P* = 0.002)
Koskinas, et al. [69]	2024	With near-infrared spectroscopy, observed attenuated reduction in maxLCBI_4mm_ with alirocumab + statin in post-MI patients with baseline elevated Lp(a) compared to those with baseline low Lp(a) (Q1–Q3, < 98 nmol/L; *n* = 99; −40.2 [− 91.1 to 10.7] versus − 91.4 [− 113.9 to − 68.9], respectively; *P* = 0.01). No significant difference between reductions in percent atheroma volume among post-MI patients with baseline high Lp(a) vs. low Lp(a)

*Lp(a)* Lipoprotein(a), *CCTA *Coronary Computed Tomography Angiography, *CT* Computed Tomography, *CAC* Coronary Artery Calcium, *HRTCFA* Thin-Cap Fibroadenoma, *LDL-C* low-density lipoprotein cholesterol, *FCT* fibrous cap thickness, *IVUS* Intravascular Ultrasound, *LAP* Low-Attenuation Plaque, *maxLCBI*_*4mm*_maximum lipid core burden index within 4 mm, *MI* myocardial infarction

## Lp(a) and Traditional Risk Assessment Tools

Traditional cardiovascular risk factors and scoring systems, such as LDL-C, blood pressure, diabetes status, or ASCVD risk calculators, may underestimate risk in patients with high Lp(a) for several reasons: (1) Lp(a) is typically not included in risk calculators, and (2) high Lp(a) creates residual CV risk even when LDL-C and other traditional CV risk factors are controlled [20, 21]. In susceptible individuals, Lp(a) is elevated starting at birth and increases throughout early childhood until reaching levels by age 5 that will remain relatively stable into adulthood [22, 23]. Thus, vascular inflammation and atherosclerosis can develop earlier, something not considered in risk calculators, quantified in other laboratory markers, or frequently considered in current clinical practice.

Understanding the various manifestations of Lp(a) on imaging is critically important because it allows clinicians to effectively account for this potential underestimation of risk. If we are able to detect the unique imprint of Lp(a) using vascular imaging, we may gain a tool to visualize the risk that laboratory data and scoring metrics fail to capture. Imaging has the potential to enhance patient risk-stratification and treatment decisions, which will become increasingly important as Lp(a)-lowering therapies are developed over the next decade. Imaging biomarkers additionally provide measurable endpoints for research, including clinical trials, allowing researchers to assess how these therapies impact the progression of plaque.

## Established Imaging Modalities

### Coronary Artery Calcium (CAC) Scoring

CAC scoring uses a non-contrast, cardiac-gated CT scan to detect coronary artery calcification. It has been validated as a tool for risk stratification, as the presence of CAC is associated with increased risk of major adverse cardiovascular events, while a CAC score of 0 is associated with low short-term risk of events [24]. As outlined in a 2025 review from Palanisamy et al., the relationship between elevated Lp(a) and CAC has not been clearly elucidated, with many inconsistencies noted between study populations [18]. While a few investigations from the early 2000s found that there was no association between elevated Lp(a) and CAC burden, more recent studies suggest that a relationship does exist [25–27].

Studies from the Multi-Ethnic Study of Atherosclerosis (MESA) and Mediators of Atherosclerosis in South Asians Living in America (MASALA) cohorts from 2021 to 2023, respectively, demonstrated a linkage between Lp(a) and progression, but not overall burden, of CAC. Garg et al. found in the MESA that increased levels of Lp(a) were associated with more rapid CAC progression, but that no significant association was present with CAC prevalence [28]. Ong et al. used MESA data to demonstrate that the association between elevated Lp(a) and an absolute increase in CAC volume was even stronger among participants who had high levels of inflammatory and coagulation markers [29]. Shah et al. demonstrated in the MASALA study that among those with CAC progression, Lp(a) > 50 mg/dL was associated with a 75% higher rate of annual CAC progression compared to those with lower levels, although Lp(a) was not significantly associated with CAC progression among the entire cohort [30]. Another study using these data found that Lp(a) was associated with progression of CAC density, but not volume, among those with progression in CAC [31].

In 2024, Wong et al. used a pooled cohort of three U.S. prospective studies to examine the effects of Lp(a) on CAC progression among different demographics. Higher Lp(a) levels were linked with greater progression of CAC among female, male, Black, and non-Black individuals, with the most significant absolute CAC progression observed in men and in non-Black individuals [32]. Another comparative study from Jackson et al. showed that Lp(a) was modestly associated with both CAC volume and progression in patients under 62 years old, but only with CAC progression when participants of all ages were included [33]. However, a large, prospective study using data from the Atherosclerosis Risk in Communities (ARIC) cohort showed that those with elevated Lp(a) were, in fact, more likely to have elevated CAC burden [34]. It was also demonstrated in another cohort that Lp(a) was associated with not only CAC progression, but also overall burden [35].

Multiple meta-analyses have shed light on this relationship when it comes to different populations. Each study entailed different baseline patient characteristics; one involved asymptomatic individuals with known cardiovascular disease, another with asymptomatic subjects without known CAD, and a third with both subjects who were symptomatic and asymptomatic [36–38]. All three found that Lp(a) levels ≥ 50 mg/dL were associated with higher likelihood (31–58%) of having elevated CAC, although the relationship between mid-range Lp(a) (20–50 mg/dL) and CAC varied.

Another 2025 longitudinal study of 41,929 patients by Sung et al. arrived at different conclusions. It was shown that different Lp(a) quintiles were not associated with incident CAC or with CAC progression [39]. It is important to note that this study was limited by the fact that many participants were young and healthy men with low Lp(a) and 10-year ASCVD risk, limiting the external validity, as discussed in an editorial by Razavi et al. [19].

Other studies have looked further into the relationship between Lp(a) and CAC. It was shown in a cohort of both MESA and the Dallas Heart Study (DHS) that Lp(a) and CAC are independent risk factors for ASCVD. Interestingly, nearly half of patients with elevated Lp(a) had a CAC score of 0. While Lp(a) and CAC were independent predictors of risk, the highest risk for ASCVD was in subgroups with both elevated Lp(a) and the presence of calcified plaque by CAC scoring. Notably, the observed 10-year ASCVD risk of elevated Lp(a) with CAC score of 0 was 3.4%, while that of elevated Lp(a) with CAC score ≥ 100 was 22.0% [40]. In a similar 2026 study using a larger sample of multiple, pooled cohorts, Bhatia et al. demonstrated that elevated Lp(a) was linked to greater relative ASCVD risk in middle-aged individuals across CAC strata, including in participants with CAC of 0 [41]. Among those with CAC = 0, it was shown that incidence of ASCVD was higher in those with Lp(a) > 50 mg/dL compared to those with Lp(a) ≤ 50 mg/dL, although absolute event rates remained low. An association between Lp(a) and increased risk was also present in patients with CAC > 0, with the greatest overall risk found in those with both CAC ≥ 300 and Lp(a) > 50 mg/dL. These findings suggest that CAC is a useful tool for risk stratification of middle-aged individuals, but further study of younger populations is needed in the future.

Thus, while there are differences in the results of studies examining the relationship between Lp(a) and CAC, data overwhelmingly suggest that a relationship between Lp(a) levels and CAC exists. Mixed findings may reflect differences in study populations, variability in Lp(a) thresholds and measurement assays, and distinct pathobiological roles of Lp(a). Specifically, Lp(a) may be more reflective of active atherogenic processes like higher risk, non-calcified plaque—reflected in imaging findings such as low-attenuation plaque formation—than of calcified plaque burden, which often represents a later and potentially more stable stage of atherosclerosis. The IMAGE-LPA prospective case-control trial will examine early manifestations of Lp(a) on CCTA and CAC among a population with a strong family history of elevated Lp(a) levels, stratifying subgroups by Lp(a) ≥ or < 150 nmol/L [42].

### Coronary Computed Tomography Angiography (CCTA)

CCTA possesses high diagnostic accuracy for detection of obstructive coronary stenosis and is widely used to rule out obstructive coronary artery disease with a demonstrated negative predictive value of 99% [43]. Lp(a) was first shown to be independently associated with the presence of coronary lesions on CCTA as early as 1986 by Dahlen et al., a relationship that has been clarified and expanded upon by advances in CT over the last four decades [44]. In cross-sectional studies, higher Lp(a) is consistently associated with greater prevalence of any type of coronary plaque [45–48]. A relationship between Lp(a) and multi-vessel disease has also been demonstrated in multiple studies [46, 49].

**Fig. 1:**
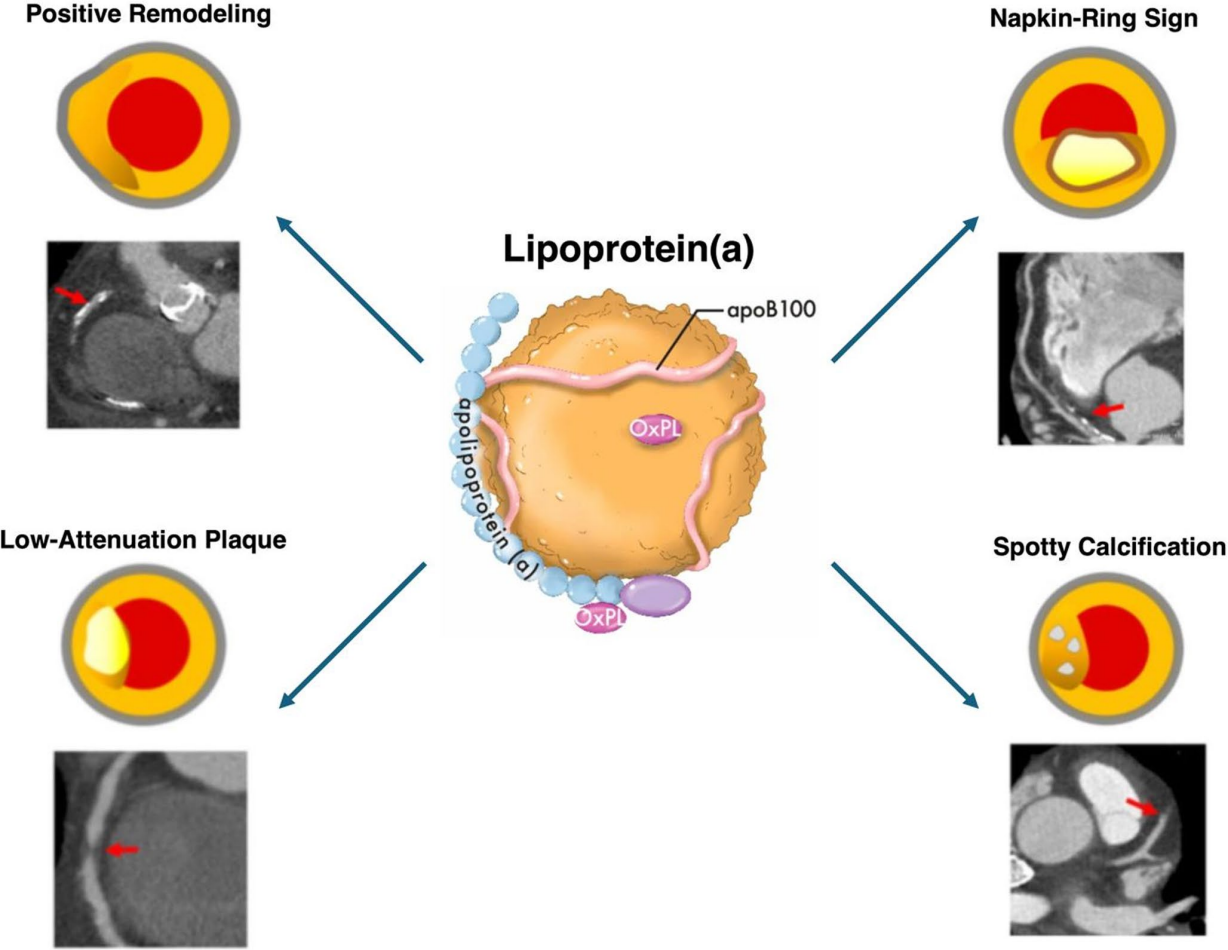
High-risk plaque features associated with lipoprotein(a), as seen on coronary computed tomography angiography. Certain morphological (high-risk) features of coronary plaque are associated with a greater risk of adverse cardiovascular events, including positive remodeling, napkin-ring sign, low-attenuation plaque, and spotty calcification. Positive remodeling represents an outward expansion of vessel area and napkin-ring sign represents an area of low-attenuation plaque with a high-enhancement rim. Central Lp(a) graphic adapted from Bhatia, et al. [52], vessel graphics and CT images adapted from Pugliese, et al. [53]

In addition to assessing stenosis caused by atherosclerotic plaque in the coronary arteries, CCTA is often used to quantify the extent of calcified, mixed, and non-calcified plaque and examine characteristics of high-risk plaque, including napkin ring sign, positive remodeling, spotty microcalcifications, and low attenuation plaque (LAP) (Fig. 1) [50]. It has been well-documented over the last decade that Lp(a) is linked to not only obstructive CAD by CCTA, but also high-risk plaque features [45, 48, 51]. Fathieh et al. recently demonstrated from 1718 participants in the BioHEART study that Lp(a) is strongly associated with mixed plaque burden after adjustment for traditional risk factors, without a significant relationship with calcified or non-calcified plaque burden alone [46]. Furthermore, the Miami Heart Study found that elevated Lp(a) was strongly associated with the presence of ≥ 2 high-risk plaque features with an OR of 3.57 [95% CI, 1.79–7.10]. It also showed that among those with a CAC score of 0, a higher percentage of any plaque was found in participants with elevated Lp(a) compared to those with normal Lp(a) levels. This study is currently ongoing.

Additionally, the presence of various measures of coronary artery disease (CAD) on CCTA, including multi-segment disease, multi-vessel disease, and LAP volume, increases as Lp(a) levels rise incrementally. This suggests that disease severity seen on imaging does not plateau once Lp(a) reaches a certain threshold, but persists as a continuous relationship [46, 54].

Another area of interest is the relationship between Lp(a) levels and the progression of atherosclerosis over time. Nurmohamed et al. demonstrated in a 10-year, single-center serial CCTA study, the longest interval window for a study of this type, that Lp(a) ≥ 125 nmol/L was associated with more rapid progression of total, non-calcified, and calcified coronary plaque in comparison with a group with Lp(a) < 125 nmol/L [55].

Multiple other studies have suggested that Lp(a) is not associated with accelerated progression of overall plaque burden over time, but rather with that of LAP volume [54, 56]. However, it is important to note that one of these studies evaluated only 36 patients with elevated Lp(a), and the other involved a cohort composed only of patients with diabetes mellitus. Lp(a) has also been associated with an increase in Gensini score rate of change per year, which quantifies the speed of plaque progression [57].

While the relationship with LAP has been elucidated more clearly and consistently, discrepancies in findings regarding Lp(a) and rate of progression of overall plaque burden require clarification with large, longitudinal, multi-center studies. It is also important to note that in addition to differences in study population and design, CT scanner and protocol variability across studies limits comparability of data.

### Optical Coherence Tomography (OCT)

OCT utilizes near-infrared light to scan the inside of a coronary artery during invasive angiography and is able to differentiate between different types of plaque and ensure adequate sizing of vessels before stent placement [58]. Elevated Lp(a) has been linked with higher prevalence of lipid-rich plaque, wider lipid arc, and thin-cap fibroatheroma (TCFA) on OCT [59]. These findings have since been expanded upon, with Muramatsu, et al. corroborating the relationship between elevated Lp(a) and prevalence of TCFA. During stratification by LDL-C levels, a significant association between Lp(a) and TCFA persisted for the high LDL-C group, but not for the low LDL-C group [60].

The HUYGENS trial analyzed the addition of evolocumab, a proprotein convertase subtilisin kexin type 9 inhibitor (PSCK9i) monoclonal antibody (mAb), to high-dose statin therapy on plaque composition, as evaluated on OCT, in post-myocardial infarction patients [61]. A secondary analysis demonstrated that evolocumab added to the treatment regimen resulted in lower levels of both LDL-C and Lp(a) compared to placebo in an elevated baseline Lp(a) group as well as in a low baseline Lp(a) group. Greater reductions in both minimum fibrous cap thickness (FCT) and lipid arc with addition of evolocumab were shown in the elevated Lp(a) group, but not the low Lp(a) group [62].

As a more invasive approach than CCTA or CAC scoring, the widespread clinical utility of using OCT to examine coronary plaque in individuals with elevated Lp(a) is likely limited. Nonetheless, these findings shed more light on the complex effects of Lp(a) on plaque burden, plaque phenotype, and cardiovascular risk.

### Intravascular Ultrasound (IVUS)

Another imaging modality with substantial data pertaining to Lp(a) is intravascular ultrasound (IVUS). Like OCT, IVUS is invasive and therefore more limited in terms of routine application for assessing the burden and characeteristics of coronary plaque in those with elevated Lp(a). IVUS enables direct visualization of coronary plaque burden, composition, vessel size, and arterial remodeling [63, 64]. It has proven to be especially important for evaluation of left main coronary artery disease, where angiography may underestimate disease severity [65]. Albeit in a small study of 60 patients, Hartmann et al. demonstrated a positive correlation between Lp(a) and progression of plaque plus media cross-sectional area. They also found that the mean Lp(a) of patients with plaque progression was 30 mg/dL, while that of patients without plaque progression was 14 mg/dL [66].

A meta-analysis published in 2020 further evaluated this relationship, utilizing a patient pool numbering 5,393 throughout six trials. Elevated Lp(a) was associated with a higher percent atheroma volume, although the 1.2% statistically significant absolute difference may have limited clinical or pathological importance. Risk-adjusted percent atheroma volume was also positively associated with higher Lp(a) quintiles [67].

The PACMAN-AMI trial evaluated the effect of adding alirocumab, a PCSK9i mAb, to high-dose statin therapy on the change in percent atheroma volume in post-myocardial infarction patients [68]. Data from this study was further analyzed by the baseline Lp(a) levels of participants, showing no difference in changes in percent atheroma volume and minimum fibrous cap thickness. There was, however, a significantly attenuated reduction in maximum lipid core burden index within 4 mm of 40.2% in the elevated Lp(a) group compared to the lower Lp(a) group using near-infrared spectroscopy [69]. This finding provides a possible explanation for the residual risk associated with elevated Lp(a) even when other lipid levels are optimally controlled.

## Emerging Techniques, Future Directions, and Research Priorities

The importance of understanding how Lp(a) influences cardiovascular risk will only continue to rise over the next decade, and recognizing its manifestations on different imaging modalities is crucial to wholly appreciate this relationship. Technology in advanced plaque characterization continues to improve, with AI-enhanced CCTA allowing for automated quantification of LAP, positive remodeling, and other high-risk features [70]. Photon-counting CT and the use of radiomics with deep learning will also play a role in refinement of noninvasive plaque characterization moving forward. Standardizing imaging-based biomarkers that specifically map to Lp(a)-driven vascular processes will enable researchers and clinicians to further stratify risk using these tools. While outside of the scope of this review, establishing whether molecular imaging techniques like targeted positron emission tomography (PET) tracing can reliably and consistently be used to detect early manifestations of Lp(a)-mediated vascular inflammation should also be a priority of future research. One such study used PET imaging to assess the impact of evolocumab on arterial wall inflammation in patients with elevated Lp(a), providing insights into this emerging area. It was found that in those with elevated Lp(a), evolocumab therapy resulted in large reductions in LDL-C with minimal reductions in Lp(a) and unaltered arterial wall inflammation [71].

With targeted Lp(a)-lowering therapies currently in development, imaging biomarkers have the potential to serve as endpoints in clinical trials. Trials with targeted Lp(a)-lowering therapies have consistently demonstrated the Lp(a)-lowering efficacy of these therapies, and cardiovascular outcomes trials are underway [72–75]. Understanding the potential relationship between Lp(a)-lowering therapies and progression of cardiovascular disease on imaging will be a crucial next step for research in this area. Not only will imaging serve as an endpoint, it may also be a tool to help decide which patients to prioritize for treatment with targeted Lp(a)-lowering therapies.

There are a few gaps that limit the overall generalizability of the research discussed in this review. The lack of consistent Lp(a) cutoffs between large, multi-center studies prevents much of the data from being directly comparable. Some studies set 125 nmol/L as a threshold for elevated Lp(a) in accordance with national guidelines, while others defined an elevation as the highest quartile or quintile of their participants, which in the PACMAN-AMI trial, for example, was > 98 nmol/L [68]. There also exists an underrepresentation of younger individuals, women, and those from diverse racial/ethnic groups in current literature, limiting overall generalizability.

## Lp(a) and Imaging in Practice

We find that results of vascular imaging are useful in our own practice for helping patients understand their individual cardiovascular risk, as well as in informing discussion around treatment options. Even without the standardized use of imaging in those with elevated Lp(a), whether isolated or with comorbid ASCVD or CV risk factors, we have found imaging to hold clinical value in motivating patient behavior. Our experience mirrors the well-documented phenomenon of showing patients a visual representation of a disease process increasing their desire to initiate or continue treatment, likely a result of enhancing patients’ risk perception and motivation for behavior change [76, 77]. For example, we have noticed that a discussion of individualized cardiovascular risk in the clinic, using recent CAC or CCTA images is often followed by an increased willingness to begin or intensify preventive pharmacotherapies (e.g. lipid-lowering therapies, anti-hypertensives, cardiometabolic therapies such as GLP-1 receptor agonists and related medications) and adopt a healthier lifestyle. We also find vascular imaging findings, including plaque burden or high-risk plaque features on CCTA, as well as the presence of an elevated CAC score, to be useful in risk-benefit discussions with patients around the initiation of aspirin 81 mg daily to prevent a first cardiovascular event [78]. Looking forward, we anticipate that Lp(a) will likely also play a role in risk stratification for patients with incidental findings of coronary artery calcified plaque discovered on non-cardiac imaging. For example, AI-based tools to detect coronary calcified plaque from non-gated chest CT may serve to initiate further risk stratification and treatment intensification, where Lp(a) levels and other biomarkers will be incorporated with these imaging findings to inform the next steps in preventive cardiovascular care [79]. The inverse is true as well; imaging findings have the potential to help inform treatment decisions in individuals where elevated Lp(a) is discovered but imaging suggests lower risk for a first cardiovascular event (e.g. CAC = 0).

As a greater number of individuals begin to undergo a one-time measurement of Lp(a) levels in accordance with contemporary guidelines, it is becoming more important that we have the tools and knowledge to interpret these results, stratify overall risk, and take action to reduce cardiovascular risk in these individuals [80, 81]. Lp(a) should be incorporated into a comprehensive, personalized assessment of cardiovascular risak and vascular imaging will have a greater role in this process as the use of these imaging modalities and understanding of their predictive abilities for risk continues to expand.

## Conclusions

Across coronary artery imaging modalities, elevated Lp(a) is linked to greater atherosclerotic plaque burden and prevalence of high-risk plaque features. CCTA currently offers the most detailed and reproducible noninvasive characterization of Lp(a)-related coronary plaque, demonstrating strong associations with overall plaque prevalence, multivessel involvement, and high-risk morphologies such as low-attenuation plaque, positive remodeling, and napkin-ring sign. However, it does carry the downsides of radiation exposure as well as expense. Longitudinal CCTA studies suggest that Lp(a) may be associated with accelerated progression of mixed and lipid-rich plaque, though results differ based on baseline risk and study design. While recent studies suggest that high Lp(a) is associated with greater CAC progression and, in some settings, higher baseline burden, a substantial fraction of individuals with elevated Lp(a) have a CAC score of zero, perhaps a reflection of Lp(a) driving earlier, non-calcified or mixed plaque vs. later and potentially more stable forms of atherosclerosis, as well as individual-level heterogeneity in CV risk among those with high Lp(a). OCT data also support these conclusions, with clear evidence supporting the relationship between Lp(a) and multiple plaque features, most predominantly TCFA. IVUS reinforces these insights by demonstrating modest correlations between elevated Lp(a) and greater atheroma volume and progression over time.

Considered together, invasive and noninvasive imaging indicate that Lp(a) influences both the burden and characteristics of coronary plaque and increases the likelihood of high-risk plaque features. Differences across studies underscore the need for standardized endpoints, longitudinal designs, and careful stratification by baseline risk to fully clarify how Lp(a) manifests across the atherosclerotic cardiovascular disease risk continuum. While imaging evidence of coronary artery plaque in those with elevated Lp(a) can be used now as part of CV risk stratification, future research should include studies to advance our ability to standardize risk stratification and treatment decisions using these imaging modalities in our patients with elevated Lp(a).

## Key References

- Martignoni FV, RL Júnior JE, Marques IR, Gomes C, Moreira VCS, de Souza IAF, et al. The association of lipoprotein(a) and coronary artery calcium in asymptomatic patients: a systematic review and meta-analysis. European Journal of Preventive Cardiology. 2024;31(6):732 − 41. doi: 10.1093/eurjpc/zwae043.
  - ○ Meta-analysis describing the association between Lp(a) levels and CAC in asymptomatic patients without established ASCVD, demonstrating a strong, positive correlation between Lp(a) levels and CAC.
- Qiu Y, Hao W, Guo Y, Guo Q, Zhang Y, Liu X, et al. The association of lipoprotein (a) with coronary artery calcification: A systematic review and meta-analysis. Atherosclerosis. 2024;388:117405. doi: 10.1016/j.atherosclerosis.2023.117405.
  - ○ Meta-analysis that examined the relationship between Lp(a) levels and CAC among all patients, finding that elevated Lp(a) was associated with a higher prevalence and progression of CAC.
- Bhatia HS, Fan Y, Dharmavaram G, Razavi AC, Tsai MY, Ramsis M, et al. Use of Coronary Artery Calcium Scoring in Individuals With Elevated Lipoprotein(a): A Multicohort Study. J Am Coll Cardiol. 2026. doi: 10.1016/j.jacc.2026.02.5067.
  - ○ Pooled cohort study of 11,319 participants that built upon the findings from MESA and DHS by using additional cohorts. This greater sample size further served to both corroborate previous insights about CAC and Lp(a), some of which had not previously been shown to be statistically significant, as well as expand on the association in a wider age range of participants.
- Mszar R, Cainzos-Achirica M, Valero-Elizondo J, Lahan S, Al-Kindi SG, Quispe R, et al. Lipoprotein(a) and Coronary Plaque in Asymptomatic Individuals: The Miami Heart Study at Baptist Health South Florida. Circ Cardiovasc Imaging. 2024;17(7):e016152. doi: 10.1161/circimaging.123.016152.
  - ○ Using data from the Miami Heart Study, this study analyzed the association between Lp(a) and plaque characteristics on CCTA in asymptomatic adults without clinical cardiovascular disease. Higher levels of Lp(a) were found to be independently correlated with the presence of plaque.
- Fathieh S, Tang O, Gray MP, Zanchin C, Vernon ST, Genetzakis E, et al. Evaluating the role of lipoprotein(a) in enhancing risk stratification for the presence and extent of subclinical coronary artery disease burden: a BioHEART-CT study. European Journal of Preventive Cardiology. 2025. doi: 10.1093/eurjpc/zwaf323.
  - ○ Data from the BioHEART study was used to evaluate the relationship between Lp(a) and multiple measures of plaque burden, quantifying CCTA findings as Lp(a) levels rose in increments of 10 nmol/L. A strong association was demonstrated between Lp(a) and all CTCA measures of CAD examined.
- Nurmohamed NS, Gaillard EL, Malkasian S, de Groot RJ, Ibrahim S, Bom MJ, et al. Lipoprotein(a) and Long-Term Plaque Progression, Low-Density Plaque, and Pericoronary Inflammation. JAMA Cardiology. 2024;9(9):826-34. doi: 10.1001/jamacardio.2024.1874.
  - ○ Single-center prospective cohort study investigating how Lp(a) levels relate to plaque progression and morphology over a 10 year interval. It was found that higher Lp(a) levels correlated with increased progression of plaque burden, presence of low-density non-calcified plaque, and pericoronary adipose tissue attenuation.
- Di Giovanni G, Fujino M, Kataoka Y, Butters J, Hucko T, Puri R, et al. Impact of evolocumab on plaque phenotypic changes in patients with acute coronary syndrome and elevated lipoprotein(a) levels: a HUYGENS secondary analysis. Eur J Prev Cardiol. 2025. doi: 10.1093/eurjpc/zwaf211.
  - ○ A secondary analysis of the HUYGENS trial, this study described the effects of evolocumab on Lp(a) levels and features of coronary plaque stabilization, as seen on OCT. The abilility of evolocumab to effectively promote coronary plaque stabilization was found to be more pronounced in patients with elevated Lp(a), indicating that Lp(a) may be useful in identifying those who will benefit the most from therapy with evolocumab.
- Koskinas KC, Häner J, Ueki Y, Otsuka T, Lonborg J, Shibutani H, et al. Association of Lipoprotein(a) With Changes in Coronary Atherosclerosis in Patients Treated With Alirocumab. Circ Cardiovasc Imaging. 2024;17(11):e016683. doi: 10.1161/circimaging.124.016683.
  - ○ This study used data from the PACMAN-AMI trial to evaluate the effect of alirocumab on IVUS, OCT, and near-infrared spectroscopy endpoints in patients with elevated and normal Lp(a) levels. Elevated baseline Lp(a) was associated with attenuated plaque regression following treatment with alirocumab and a high-intensity statin, compared to those with normal Lp(a) levels.

## Data Availability

No datasets were generated or analysed during the current study.
